# Primary Immunodeficiency and Cancer Predisposition Revisited: Embedding Two Closely Related Concepts Into an Integrative Conceptual Framework

**DOI:** 10.3389/fimmu.2018.03136

**Published:** 2019-02-12

**Authors:** Oskar A. Haas

**Affiliations:** Department of Clinical Genetics, Children's Cancer Research Institute, Vienna, Austria

**Keywords:** primary immunodeficiency, cancer predisposition, down syndrome, childhood leukemia, immune editing, immune activation, inflammation, microbiome

## Abstract

Common understanding suggests that the normal function of a “healthy” immune system safe-guards and protects against the development of malignancies, whereas a genetically impaired one might increase the likelihood of their manifestation. This view is primarily based on and apparently supported by an increased incidence of such diseases in patients with specific forms of immunodeficiencies that are caused by high penetrant gene defects. As I will review and discuss herein, such constellations merely represent the tip of an iceberg. The overall situation is by far more varied and complex, especially if one takes into account the growing difficulties to define what actually constitutes an immunodeficiency and what defines a cancer predisposition. The enormous advances in genome sequencing, in bioinformatic analyses and in the functional *in vitro* and *in vivo* assessment of novel findings together with the availability of large databases provide us with a wealth of information that steadily increases the number of sequence variants that concur with clinically more or less recognizable immunological problems and their consequences. Since many of the newly identified hard-core defects are exceedingly rare, their tumor predisposing effect is difficult to ascertain. The analyses of large data sets, on the other hand, continuously supply us with low penetrant variants that, at least in statistical terms, are clearly tumor predisposing, although their specific relevance for the respective carriers still needs to be carefully assessed on an individual basis. Finally, defects and variants that affect the same gene families and pathways in both a constitutional and somatic setting underscore the fact that immunodeficiencies and cancer predisposition can be viewed as two closely related errors of development. Depending on the particular genetic and/or environmental context as well as the respective stage of development, the same changes can have either a neutral, predisposing and, in some instances, even a protective effect. To understand the interaction between the immune system, be it “normal” or “deficient” and tumor predisposition and development on a systemic level, one therefore needs to focus on the structure and dynamic functional organization of the entire immune system rather than on its isolated individual components alone.

## Introduction

The neoplastic transformation of cells and their subsequent successful clonal expansion and progression into clinically apparent hematologic malignancies and solid tumors is a complex multifactorial process. On the one hand, this process requires changes in the respective cells' genetic program that modify their metabolism and performance and consequently alter their normal differentiation, replicative, and survival capacity. On the other hand, these cells have to learn to adapt themselves and to exploit external deterministic physiological stimuli as well as to flexibly react to a plethora of stochastic environmental challenges (1, 2). This, in turn, defines their capability to achieve successful interactions with and survival strategies within their normal surrounding tissue. With its interactive network of cells, humoral factors, and cytokines, the immune system plays a fundamental role in the recognition of and protection against any internal or external threads, be it abnormal cells, foreign tissues or infections agents. Inborn genetic defects or dysfunctions of the one or the other immune system components may thus unsettle the intricate physiological balance and maintenance of a body's functional homeostasis and thereby diminish its preventive capability or even promote the formation of neoplastic diseases in a proactive manner.

The recent methodological advances in deciphering the composition and structure of the human genome allow us now to identify virtually any DNA sequence alterations in a hitherto unimaginable fast and detailed manner. Various such technologies have in the meantime become invaluable diagnostic mutation screening tools that help to identify clear-cut disease-associated genetic defects in inborn errors of the immune system but also more elusive variants that may participate in the predisposition to malignant diseases in children. These developments are addressed in a large number of original publications as well as in many excellent reviews of these subjects (3–16). Rather than reiterating what has already extensively been written about, I intend to provide a more conceptional framework of this subject and focus my attention on often neglected and less well-appreciated fundamental facts and phenomena, which I consider particular relevant for an in-depth appreciation and understanding of this topic and which I will supplement with some specific examples that illustrate the developments and progress in this field.

To begin with, we first need to (re)define the current view and understanding of “primary immunodeficiency” as well “genetic predisposition and susceptibility.”

## Primary Immunodeficiency Syndromes (PID)

The immune system is composed of highly specialized cells, tissues, organs and soluble factors that interact in a complex way to ensure an organism's immune defense. According to the current definition, PID are thus a group of diseases, which are caused by heritable DNA sequence alterations that impair the quantitative or qualitative function of cellular or humoral components of the adaptive or innate immune system (17). The spectrum of their clinical, often intimately interrelated symptoms includes developmental disorders, autoinflammation, chronic inflammation, autoimmunity, neoplasms as well as serious, recurrent, or unusual infections (18, 19). Initially, the diagnosis of these conditions was based on abnormal laboratory parameters and clinical problems, in particular recurrent, severe or unusual infections that in certain groups of patients occasionally concurred with familial clustering, syndromic features, radiation sensitivity and also a certain propensity to develop particular types of malignancies. With the advent of *in vitro* testing and immunophenotyping technologies, it became possible to better define and differentiate certain categories as well as to characterize even subtle cellular and humoral functional deviances already to a certain extent. In the early days of the molecular genetic era, the respective responsible genes were then identified in cases with highly penetrant genetic traits, which instigated a first, albeit restricted diagnostic mutation screening. With the introduction of more sophisticated sequencing technologies, the discovery of causative genetic defects increased steadily in parallel with the refined dissection, delineation, and definition of such immunodeficiency syndromes. The recent 2017 update of the “Primary Immunodeficiency Committee” of the “International Union of Immunological Societies” thus recognizes 344 genetic defects that define 354 distinct disorders of immunity in nine categories (20, 21). Some of these monogenetic disorders are extremely rare and were so far identified in single families only.

This compilation together with the commonly unconsidered use of the term PID leaves the impression that one indeed knows what the term PID stands for. It is therefore intriguing to note and especially important to point out that there is actually no clear consensus about its definition (22). The reason for this now newly flaring-up debate is the recognition that the perception of immunodeficiency has so far clearly focused only on the most obvious and clinically striking disorders in both adaptive and innate immunity that affect the lympho- and hematopoietic system. With the increasing appreciation that also non-hematopoietic cells and tissues participate in a significant manner in the immune defense this view is currently changing and necessitates an expansion of this concept. For instance, keratinocytes, endothelial cells, and fibroblast secrete as much and as many cytokines as hematopoietic cells do and can thus use their intrinsic pathways for protection against infectious agents also in a similar fashion. Another example are neurons and oligodendrocytes, which are similar essential and sufficient guardians against herpes simplex virus I and probably also other infection agents (22).

Another development that one has to consider in this context are the results that derive from the increasingly sophisticated diagnostic work-up of suspicious cases with technologies that enable nowadays the recognition of even clinically not readily apparent quantitative and qualitative deviations of particular cellular and humoral immune system components. As can be appreciated already in a normal setting, such differences are commonly due to and thus correlate with variations on the sequence level, either in form of single nucleotide polymorphisms/(SNP) alone or in form of definable haplotypes, which can make it more and more difficult to define a physiological norm and, under particular settings, a clear disease-relevant pathological state (23–32). One of the best documented and therefore most instructive example is the context-dependent implications of the highly variable serum levels of the mannan-binding lectin (MBL), the apparently most common deficiency of a humoral component of the innate immune system (33). The respective gene contains 87 different polymorphic sites with a multitude of possible combinations, of which seven common haplotypes stand out. These haplotypes determine the serum levels as well as the configuration and function of the encoded proteins in a predictable manner, although the possible effects are co-determined by the sex and age of the respective carrier, hormonal changes and immune system activation. Moreover, the frequency of the diverse haplotypes varies world-wide in an ethnicity specific manner. Thus, although the magnitude of particular MBL protein levels are clearly recognizable and determined by genetic factors, the ensuing effects, whether a low or high level becomes detrimental or beneficial or whether it remains irrelevant, are strictly context-dependent and therefore difficult to predict or interpret in a given individual.

Based on an estimate that ~5% of all genes participate in one or the other way in host defense and immune tolerance, it was predicted that with the new sequencing technologies up to 3,000 PIDs will be identified by the year 2021. Even if one considers only a still monogenic scenario with only two types of alleles per locus (i.e., heterozygous vs. homozygous, loss-of function vs. gain-of-function, hypomorphic vs. amorphic as well as various other variations), it is hardly imaginable that one will be able to functionally evaluate and analyze the magnitude of all the possible outcomes in a reasonable manner to make some sense of the ensuing (patho)physiological effects even in an perhaps otherwise well-defined setting (22, 34).

In the end, these reflections leave us with the question how one actually will define primary immunodeficiencies in the future. When, to which extent and in which form do they need to manifest themselves clinically? Will it be sufficient to just view them as pure monogenic disorders or does one eventually also need to consider the contribution of modifying gene, signaling pathway, and cellular networks in a much stronger way?

## Genetic Predisposition and Susceptibility

The concept of genetic predisposition and susceptibility, which so far was also based primarily on the clinical perception of disease and inheritance patterns, experiences nowadays a similar reinterpretation and paradigm shift as the one of immunodeficiency. The emergence and continuous improvement of fine-scale and cost-efficient targeted, whole exome and whole genome, methylation as well as RNA sequencing approaches, increase the possibilities to investigate the genetic background of heritable and acquired diseases in a previously unprecedented manner (6, 10). Not only has it become much easier to screen all the eligible genes of already well-recognized conditions for causative mutations, it has also become much simpler to identify novel sequence abnormalities in rare, unusual, or merely suspected cases of immunodeficiencies and cancer predisposition. Thus, the special choice of the appropriate mode to search for and ascertain such genetic factors remains nowadays a matter of intention, clinical opportunities and individual demands that, in particular, is based on patient/family-relevant, gene-related, disease-associated, or population-based aspects (30). In a clinical setting, the direct patient-orientated approach is definitely the most important one. The ascertainment of an inborn genetic cause of a particular disease requires not only its appropriate work-up with the best-fitted mutation screening method but also the careful justification of its significance through the assessment of medical records and family history as well as the clinical and laboratory data of an affected patient (7, 13, 14, 34–47). Especially in those cases in which a cancer-prone condition is recognized already before the onset of a malignant disease, securing the specific genetic cause is essential to guide the necessary clinical measures, such as an appropriate treatment and surveillance program together with a suitable genetic counseling (7, 13, 14, 34–47). However, in many instances a potential predisposing germ-line alteration may only be suspected and searched for at the time a malignant disease is diagnosed. Especially if one screens the neoplastic tissue for disease-specific diagnostic alterations, one cannot avoid coming across inborn genetic errors, not only those in already known genes but occasionally also in novel ones. Distinguishing somatic from inherited defects in tumor tissue alone may turn out quite difficult because both types often affect the same genes, a fact that necessitates the verification of the inborn nature of any such changes by analyzing germ line material in addition. Based on such experiences, it is therefore becoming practice to screen for underlying germ-line defects in a more systematic fashion in form of so called “trio analyses,” which in addition to a patient's tumor and germ line DNA also requires the parental ones for comparison (4, 13, 34, 35). An increasing number of publications confirm that this approach is particular rewarding, not only for the sake of the patient and her family but also for scientific reasons. In case a particular gene defect is not already clearly indicative of a specific type of predisposing condition, it may be difficult or virtually impossible to decide whether concomitant immune system derangements at diagnosis are actually the cause or the effect of the respective disease. As I will point out later, more subtle predisposing gene alterations that merely modify the function of a gene, such as single nucleotid polymorphisms (SNPs), may not even exert any easily recognizable effects prior to onset of the malignant disease. Predisposing SNPs were originally discovered by large-scale genome wide association studies (GWAS) in regions of the genome, which are linked with particular disease traits. The biological relevance and functional consequences of some of these variants has in the meantime already been established and confirmed with appropriate experiments and test systems (23, 26, 48, 49).

Our current knowledge of the genetic basis of immunodeficiency and tumor predisposition is primarily based on monogenic disorders. We learned to appreciate the *genetic heterogeneity* of these conditions, meaning that single or similar phenotypes can be generated by different genetic mechanisms. *Polygenic* diseases, on the other hand, are caused by the joint contribution of several independent acting or interacting genes, whose individual contribution might be small or even unnoticeable. GWAS together with WGS studies have now allowed us to extend such analyses to the entire genome in a kind of *omnigenic* approach, which means that we will need to learn to cope with the combinatorial effects of a large number of genetic variants, whose individual contribution is not readily apparent (50). In contrast to Mendelian diseases, which are primarily caused by mutations in the protein-coding part of the genome, complex traits are mainly driven by non-coding variants that presumably affect regulatory elements of genes, such as promoters and enhancers. For instance, risk variants for autoimmune diseases show particular enrichment in active chromatin regions of immune cells (51–53).

The “*omnigenic*” model still accepts that only a modest number of “core genes” or pathways are etiologically important for a specific disease and their dysfunction will still have the strongest impact on the disease process (54). However, in this situation the particular risk will be driven by an accumulation of weak and heterogeneous effects of many modifying gene variants, whose specific configuration might even only become relevant in certain cell types and tissues, whereas in others they might remain completely inconsequential (51). The ultimate and most provocative conclusion and interpretation of this “*omnigenic*” model is of course that virtually any variant with regulatory effects in a given tissue is likely to have some (weak) effects on all diseases that are modulated through this particular tissue (51).

**Figure 1:**
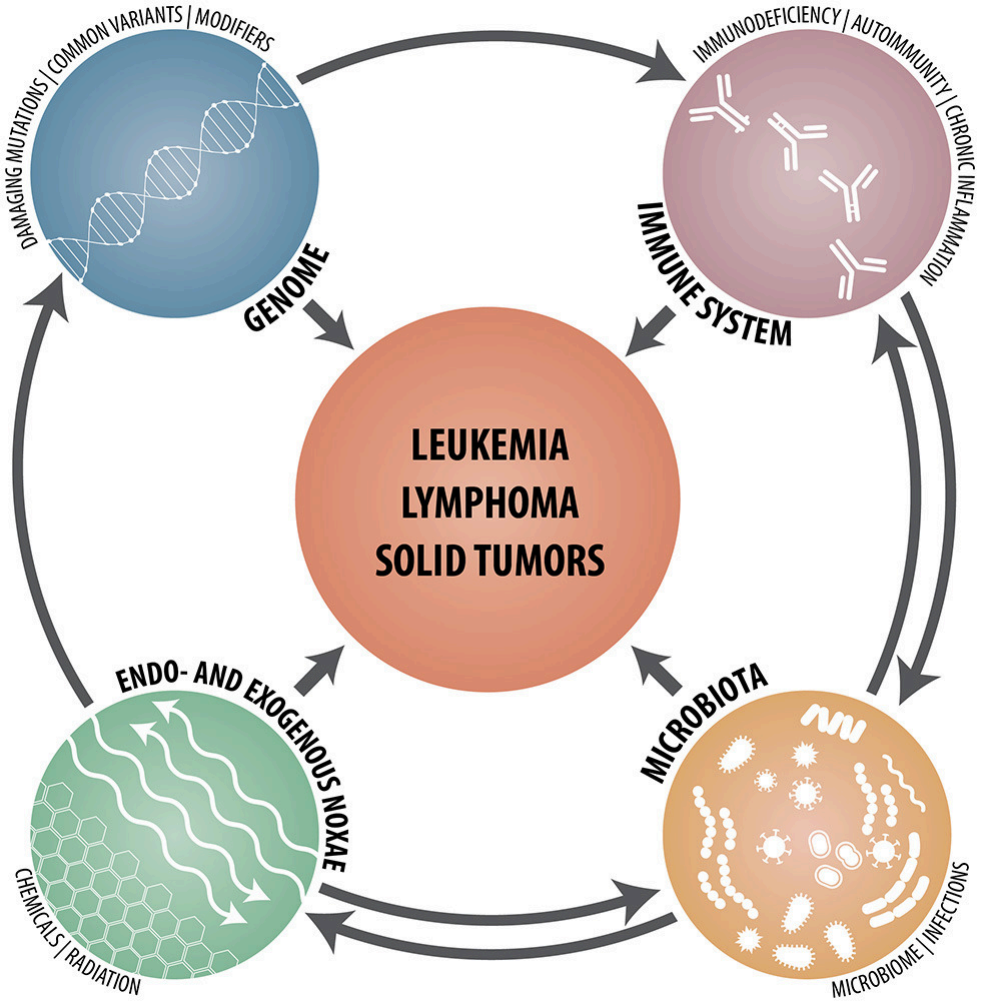
Schematic synopsis of the various genetic, immunological, microbial, and environmental constituents that contribute to and participate in the development of hematologic neoplasms, lymphomas and solid tumors.

Whereas the identification of risk factors in monogenic diseases requires sequencing of specific genes and the careful functional assessment of any unusual sequence variant that pops up, polygenic risk scores of common diseases are statistically determined likelihoods that are calculated from genome-wide SNP patterns. Given the countless possibilities how defective and normal but functionally dissimilar allele variants of one or multiple genes can be combined and co-inherited, it is therefore astonishing that, as reported recently by Khera et al., the risk scores of such common diseases may under particular circumstances nevertheless reach at least the same magnitude as the ones achieved in monogenic diseases (55). Together with the cell- and tissue-specific utilization of the ensuing gene products, these findings provide a ready explanation for the highly variable penetrance of genuine gene defects and, even more so, for functionally modifying variants and, not least, why it is so difficult to foresee their biological consequences even in monogenic disorders (34). In addition, one has of course also to keep in mind that even in instances with a strong predisposing genetic component, the development of malignancies is always a multifactorial process that not only requires a liable genetic architecture but also some probabilistic elements as well as the participation and interaction of a multitude of other intrinsic and extrinsic factors and mechanisms. In case of hematologic malignancies, such cell-intrinsic defects and abnormalities consist of those that affect (I) (lympho- and hematopoietic stem) cell development, differentiation and apoptosis; (II) lymphocyte signaling, cytoskeleton, cytotoxicity and metabolism and (III) chromosome stability as well as DNA repair (3). Cell-extrinsic factors, on the other hand, comprise chronic inflammation; autoimmune- and autoinflammatory diseases, chronic (viral) infections and an impaired tumor surveillance (Figure 1).

## Somatic Mutation (SMT) vs. Tissue Organization Field (TOFT) Theory

SMT and TOFT are two apparently competing theories of cancer development. The SMT postulates that cancer is a molecular, gene-based disease that derives from single cells whose autonomous and unrestrained proliferation is driven by the progressive accumulation of accidental and essentially unrelated events (2, 56–58). The TOFT, on the other hand, posits that cancer develops as an adaptive, emergent phenomenon whose fundamental determinants act at the level of tissue and organ homeostasis. In this scenario, inherent genetic constituents as well as a variety of physical, chemical and biological agents, such as cytokines, viruses, chemicals and/or radiation, perturb the functional interaction of diverse cellular modules and subsequently also the organizational state of tissues themselves (58). To a certain extent this process resembles morphogenesis and as such also replicates a tumor's capability to continuously balance novelty with stability and to combine plasticity with robustness (57, 59). The reductionist, bottom-up approach of SMT and the emergentist, top-down approach of TOFT are often considered incompatible because they view the problem from two different levels of biological complexity. The probably smallest and already generally agreed-upon common centerpiece where these two opinions meet is the tissue micro-environment (57).

Compared to the possibilities of compact tissues, the various closely interconnected humoral and cellular components of the immune system are in a unique situation, because they can exert their action not only in a local microenvironment, but they can also act over and cover the macroenvironment of tissues, organs and even an entire organism in a systemic fashion. Moreover, within a particular context and a respective cellular milieu, components of the immune system can either foster or suppress tumor development. It is thus not surprising that the highly flexible and adaptable immune system, be it normal, impaired, or defective, is one of the major players in the game of tumor predisposition and development (60–64).

The three prevalent and often closely connected complications of PIDs are thus infections, autoimmunity, and malignancies. Nevertheless, it is intriguing to note that except for a few distinct disorders, such as Nijmegen breakage syndrome (NBS), Ataxia telangectasia (AT), and autoimmune lymphoproliferative syndrome (ALPS)-related autoimmune diseases, PIDs do not cluster with malignancies in the human diseaseome network (60, 65). Most of the available information regarding cancer risk derives from specific subtypes that result from defects in genes that regulate DNA repair, cell cycle, apoptosis, bone marrow maturation as well as those that help to protect against virus infections (17, 60). As might be expected, the most common overall hitherto documented forms of malignancies in all these conditions are lymphomas, whereas other neoplasms occur predominantly in a more disorder-typical and -constrained manner (7, 14, 17, 38, 60, 66–72).

## “Environment”

Multicellular organisms are organized in a modular fashion with distinct functional units and compartments. Based on the particular level of organization one can thus distinguish various internal as well as external forms of environment. From the perspective of cells, for instance, such environmental shells may constitute specific niches, tissues, organs and the entire organism. The environment of a developing fetus, on the other hand, is provided by the mother. Following birth, the organism becomes embedded in a milieu of beneficial as well as latent pathogenic microorganism and is then openly exposed to the potentially damaging biological, physical, and chemical agents of the outer world, with which it has then to interact in various ways.

### Fetal-Maternal Immune System Interactions

With regard to immunology, pregnancy is a particular interesting condition because it requires the constructive co-existence of two genetically and immunological distinct individuals within a single body (73, 74). To succeed, this endeavor requires the beneficial cooperation of a fully developed, but during this period dampened, immune system with a just evolving one that still has to mature and achieve its independence. This interaction requires the temporary reorganization and adaptation of the maternal immune system as well as the acceptance of assistance and cooperation of the fetal one. Maternal immune cells therefore help to “teach” those of the fetus to balance the need of self-defense against that of immune tolerance: too much restraint would lead to lethal infections, whereas too little would lead to autoimmunity (75–77). During this especially vulnerable phase this intricate balance can easily be disturbed, in particular by both cell intrinsic (genetic) as well as cell extrinsic biological factors.

Although a fetus expresses genetically foreign paternal antigens, it coexists in harmony inside the mother because it resides in an immune-privileged cocoon (78). Nevertheless, maternal and fetal cells still traffic through the placenta during the entire gestation period. After birth, surviving cells become then the source of a lifelong micro-chimerism in the corresponding opposite bodies, a phenomenon that under particular circumstances may significantly impact the future life of the mother as well as the child in various positive or negative ways (78, 79). Changes in the number, phenotype or distribution of microchimeric cells, for instance, can have an effect on immune surveillance, tissue repair, autoimmune diseases and tumorigenesis. It has thus been suggested that microchimeric cells may modulate health and disease in a similar way as commensal microorganisms control the susceptibility to various immunological and non-immunological disorders (78, 80).

One of the striking pathological consequences of the bidirectional cell trafficking are particular forms of SCID, in which the accumulation of a significant number of maternal T cells can cause a kind of graft-versus-host disease (GVHD) in the immune incompetent child (81, 82). However, even asymptomatic infiltrations of maternal cells are still an independent predictor for the development of GVHD later in life in case of transplantations (82). Conversely, maternal micro-chimerism in cord blood can mediate a graft-versus-leukemia effect in cord blood transplantation (83). Finally, there are also rare instances of maternal-fetal transmissions of malignancies, such as has been reported for lymphoma, leukemia and melanoma (84).

Within the setting of such pre- and postnatal fetal/maternal immune system interactions, the human leukocyte antigen (HLA) system and, in particular, its paternal component plays a particular important role (78). Exposure to inherited paternal antigens as well as non-inherited maternal antigens during pregnancy can lead to either immunization or tolerization, the sequelae of which can have even consequences decades later, not least in form of an alloimmune response in case of transplantation (85).

### HLA System

The HLA system is part of the human major histocompatibility complex (MHC), a region on the short arm of chromosome 6 with some 260 genes that are involved in the immune response. Unsurprisingly, this is also the region of the genome that is associated with the greatest number of diseases with an immune system component, including those “bare lymphocyte” SCID disorders that are caused by deleterious mutations in certain MHC genes (86–88). As one of the main players in immune system interactions, the HLA system orchestrates the induction, regulation and fine-tuning of immune reactions and, in particular, the selection of the T cell repertoire (89). It is highly polymorphic and comprises more than 15,000 allelic variants (86).

Although specific HLA genotypes do not *per se* predispose to any particular disease in the strict direct sense, they are still highly enriched in and closely associated with distinct forms of inflammatory, autoimmune, and malignant disorders, a fact that not only underlines the central position of this regulatory system in their pathogenesis but also somehow links these otherwise disparate diseases.

HLA genotype patterns are not only associated with distinct sub-types of leukemias and lymphomas, but they can even correlate to some extent with the prognosis and survival of the respective diseases (90, 91). This evidence derived originally from the atypical HLA segregation patterns in leukemic families. They revealed an increase in HLA-identical non-affected sibs, HLA homozygosity, and identical disease-related maternal class II DRB1 haplotypes (90). Together this data is also taken as an indication that ALL is a problem that results from a population level response of HLA to infectious disease. In other words, overrepresented HLA haplotypes can provide some valuable insights into gene environment interactions as well as why and how particular clones are selected that will eventually produce the leukemic cell mass (88, 92–94). According to Greaves, B cell precursor (BCP) ALL evolves in two discrete steps, the *in utero* formation of a preleukemic clone, which is then triggered by a delayed abnormal immune response to common infections, followed by its postnatal conversion to overt leukemia (95–97). In the absence of any direct evidence for a specific causative infectious agent, the respective HLA pattern can thus be used as an indirect proxy measures for a genetically directed immune response and can thereby deliver valuable clues for the involved mechanisms. Based on such investigations, Taylor et al. concluded that BCP ALL is an indirect outcome of a transient auto-immune induced inflammatory molecular mimicry reaction that in turn may also explain why this subtype appears to be associated with delayed infection (88, 92, 93). Other factors that can help tumor cells to escape immune surveillance include somatic mutations that result in structural and functional changes in HLA system components, loss of expression of tumor antigens, lack of co-stimulatory molecules, and production of immunosuppressive cytokines (86). However, even more intriguing is the recent discovery that the HLA class I genotype is also participating in sculpting the entire oncogenic mutation landscape of a neoplasm (98). This is achieved through the continuous elimination of tumor cells with mutations that primarily produce strong antigens, which leads to a selection of cells with mutations that avoid producing such neoantigens (98).

Since the first hit that initiates the formation of the majority of pediatric cancers and leukemias occurs already very early in fetal life, the largest part of their further development still takes place *in utero* (95–97). It is therefore conceivable that fetal/maternal interactions and, in particular, the maternal immune system can influence and modify also the course of the disease in the one or the other way. Because one cannot study these processes directly, one has to rely on the above discussed traces and patterns that remain imprinted especially in the child's immune system after birth and which at least can provide some indirect clues about what had happened prenatally. The essential role of the *in utero* environment is further underlined by the fact that in individuals with a preexistent germline predisposition, secondary leukemia-promoting mutations can only evolve in special niches during distinct stages of organ development. In Down syndrome, for instance, *GATA1* mutations, which emerge exclusively during fetal liver hematopoiesis, cause an accumulation of immature erythro-megakaryocytic precursor cells (99–102). After birth, this pseudo- or preleukemic cell population is usually uncapable to maintain itself any longer and usually collapses within a short period. Although such spontaneous postnatal regressions of embryonic malignancies are quite common, it is not yet clear, whether at all or to which extent this phenomenon can also be attributed, in the strictest or in a broader sense, to the loss of the fetal/maternal interaction or altered activity of the newborns immune system (68, 103).

### Disorders of the DNA Repair System

Embryonic malignancies as well as those associated with PIDs are similar unfortunate byproducts of the complex processes that control normal development (102, 104–106). Environmental triggers such as carcinogenic pollutants and radiation play in general only a subordinate pathogenetic role in the development of childhood malignancies (105, 106). Such factors become primarily relevant only in those PID forms that are due to some types of DNA repair deficiencies (60, 71, 107–112). One of the physiological tasks of this system is to orchestrate processes, such as V(D)J recombination, class switch recombination, and somatic hypermutation, which together generate those lymphocyte-specific reorganizations that provide the basis for the adaptive immune system's genetic diversity (71, 108). Therefore, immune deficient patients with a dysfunctional DNA repair, such as those with an AT, NBS, and Bloom syndrome, are prone to develop lymphomas, whereas those with a dysfunctional DNA repair but without immune deficiency, such as xeroderma pigmentosum, Fanconi anemia, Werner syndrome, and Rothmund–Thomson syndrome, will primarily develop other forms of cancers (71). These occur especially in organs with rapidly dividing cells and/or an increased metabolic activity, including the brain, skin, breast, and the gastrointestinal tract. Since particular DNA repair defects produce characteristic mutation patterns and predispose to specific tumor forms in PID patients, it is in turn even possible to infer already from such indicators which DNA recombination processes are impaired (71).

Patients with constitutional mismatch repair deficiency (CMMRD) are prone to develop gastrointestinal, genitourinary and brain tumors, lymphomas, and leukemias (38, 39, 46, 47, 109, 113–116). They may also develop antibody deficiencies of variable severity, although they are neither a constant nor obligatory feature and usually also have no clinical correlate (107).

Genetic defects that disrupt the normal function of the DNA nucleotide excision repair (NER) complex cause at least eight overlapping phenotypes, such as xeroderma pigmentosum (XP), Cockayne syndrome (CS), and trichothiodystrophy (TTD) (117, 118). NER is responsible for fixing UV-induced lesions, bulky chemical adducts and some forms of oxidative damage (118). The respective complex comprises at least 30 proteins, three of which (XPB, XPD, and TTDA) are also part of the basal transcription factor TFIIH. The most interesting and intriguing of these proteins is XPD, because, as Lehman pointed out so suitably, it is one gene with two functions (DNA repair and transcription) and three diseases (XP, CS, and TTD) (117). Some of the clinical features of these syndromes are quite similar but some are also markedly different (119). Although all three syndromes have an exceptionally sun-sensitive skin, cancers develop only in patients with XP but not in those with CS and TTD (118). CS and TTD, on the other hand, have neurodevelopmental abnormalities and the latter also ichthyosis and brittle hair. To explain this conflicting and somehow mysterious genotype-phenotype discrepancies, Bootsma and Hoeijmakers proposed already quite some time ago that XP would result, if the defect would concern the DNA repair but not the transcriptional function of the complex, whereas, *vice versa*, the developmental TTD-associated problems would arise, if only the transcriptional part would be affected (120).

DNA repair defects may also impair the formation and production of antibodies, which is the defining feature of CVID (19, 121–127). Patients with such a dysfunctional humoral immunity have an increased probability to develop extra-nodal non-Hodgkin B cell and mucosa-associated lymphomas as well as epithelial tumors of the stomach, breast, bladder, and cervix (70, 128–130). In contrast to most PIDs, CVID-associated lymphomas are more common in older people and usually EBV-negative (70, 128–130). Selective IgA deficiency, in particular, goes along with a 7- to 10-fold increase in gastric adenocarcinomas. This risk is most likely related to an inability to clear *Helicobacter pylori* infections and appears to decrease when these bacteria are eradicated (70, 129, 130).

### Immunoediting

The immune system is in many, apparently paradoxical ways involved in the manifestation and evolution of malignancies. It can facilitate cellular transformation as well as prevent, promote, control and thus shape their development, phenomena that eventually were summarized under the term “cancer immunoediting” (62, 131–137). The concept of immunoediting evolved from the older and more controversial “cancer immune surveillance” one, which was based on the notion that, analogous to the “non-self” of pathogens, our immune system is also able to discriminate between the “malignant self” of pre-cancerous and cancerous cells and the “self” of normal cells (61, 62, 68). To discriminate cancer cells from normal cells, the immune system pursues two main strategies: T and B cells, which belong to the adaptive immune system, recognize altered self-proteins, whereas natural killer (NK) cells, gamma/delta T cells and macrophages, which are part of the innate immune system, take care of stress-induced self-molecules on transformed cells (61). Still, the necessity to establish an effective antitumor response, goes always hand in hand with the formidable challenge to circumvent the destruction of normal cells and to avoid autoimmunity.

Given the tight interaction between the immune system and neoplastic tissues, one naturally expects, and as Corthay put forward in eight arguments, that individuals with PIDs are more prone to develop tumors than the general population (61). At first sight this notion is well-supported by both animal models and clinical observations (61, 68). The best evidence that this is indeed the case derives from experiments with mice that lack key components of the immune system. They have not only an overall higher tumor incidence, but they are also more susceptible to transplanted or chemical carcinogen-induced tumors (64, 138). At second sight, however, all hitherto available data argues against the long-hold notion that potentially dysfunctional immune surveillance mechanisms indeed increase the general tumor risk in all PID patients. If at all, such processes can only play a subordinate and ancillary role.

Reliable information regarding the general and specific tumor risk of individuals with PIDs derives primarily from three large epidemiological studies from the USA, Australia, and the Netherlands (67, 128, 139). Together these studies comprise more than 5,000 patients with around 300 different forms of PIDs. Compared to the general population and previous estimates, these analyses revealed a surprisingly low, only 2-fold increased, overall tumor risk. However, since the respective risk is primarily confined to and therefore significantly higher in the nine most frequent high-penetrant PIDs, it is conversely also much lower or even absent in most of the other PIDs. Distinct genetic PID defects predispose to and concur with special and often unique types of malignancies, the most common of which are non-Hodgkin lymphomas, leukemias, digestive tract as well as virus-induced cancers (67, 68, 70, 128, 129, 139). This particular distribution patterns can already provide some important clues about the underlying defective, disrupted or impaired immune processes that trigger such disease developments. The two major driving forces that are responsible for the 8- to 10-fold excess lymphoma risk in subjects with PIDs, for instance, are a deficient DNA repair and an inadequate response to viral infections (61, 67, 68, 70). The intriguing part of this story is, however, that the incidence of the most frequent cancers, such as breast, lung, and colon, is in the remaining group of PIDs much lower than that in the general population.

### Inadequate Activation and Response of the Immune System

Chronic inflammation, autoinflammation, autoimmunity, and infection-associated overstimulation are closely intertwined derangements of a deficient or compromised immune system (140).

### Chronic Inflammation

Inflammation is a physiological response to tissue stressors such as tissue damage and infectious as well as non-infectious agents that ensures the maintenance of tissue homeostasis (141). Under normal circumstances it mitigates infections, clears damaged cells, and initiates tissue repair (142). If this process is not properly terminated, it can cause substantial collateral damage and contribute to tumor development (143–145). Chronic inflammation plays not only a pivotal role in different stages of tumorigenesis, but it may also impede the response to therapy (63, 145). Along the route to tumorigenesis, intrinsic genetic factors interact with extrinsic immune system and stromal humoral and cellular components to generate a mixed microenvironment that is composed of tumor-promoting and tumor-suppressive factors, including innate NK and adaptive T cells (62, 63, 145, 146). The fact that, despite their dissimilar etiology and physiology, autoimmune and infectious diseases take advantage of the same immunosuppressive pathways and logistics underlines the global relevance and central position of these particular activities (146).

Infection-provoked chronic inflammatory conditions predispose especially but not exclusively to the development of Hodgkin's, Burkitt's, and mucosa-associated lymphoid tissue (MALT) lymphomas (147–149). The best-known associations are those between *Helicobacter pylori* and gastric lymhomas, Chlamydophila psittaci and ocular adnexal lymphomas as well as *Borrelia burgdorferi* and cutaneous MALT lymphomas, while chronic infections with Epstein-Barr virus (EBV) usually predispose to Burkitt- & Hodgkin lymphomas, those with Hepatitis C virus (HCV) to marginal zone lymphomas and those with Hepatitis B virus (HBV) to hepatocellular carcinoma (147, 148). The pathogenetic role of chronic inflammation remains less clear in case of human papillomavirus-, herpes simplex virus 2-, and cytomegalovirus-triggered malignancies (150).

Inflammatory bowel diseases, such as Crohn's disease and ulcerative colitis, provoke especially the development of colorectal cancer (151, 152). Crohn's disease, in particular, is a multifactorial disease whose genetic underpinning encompasses 71 so far recognized susceptibility loci (31). Amongst these are the first recognized monogenic causes of such diseases, namely interleukin-10 (IL-10) and IL-10 receptor (IL-10R) loss of function mutations that are the specific cause of a severe, very early onset type of inflammatory bowel disease (153–156). Affected children have an extremely high probability to develop a unique form of monoclonal EBV-negative diffuse large B cell lymphoma, which is characterized by a constitutive activation of the NF-kappaB pathway and a defective local T cell immune response (153). These findings prompted Neven et al. to postulate that not gut inflammation itself but that the defective IL-10 pathway alone was the responsible pathogenetic trigger. Referring to the fact that all these children received azathioprine, which is a well-known lymphoma risk-increasing factor in adults with inflammatory bowel diseases, they suggested that this immunosuppressive treatment was also the final spark that ignited lymphoma development in these children (153).

### Autoimmunity

Autoimmunity is a prominent element in many PIDs, especially in CVIDs, and not least in those, which predispose to malignancies (19, 125, 157–159). Despite their close clinical and genetic interrelationship, autoimmunity, and PIDs were up to now interpreted as two mutually exclusive conditions rather than as two sides of the same coin (157). In consideration of the fact that autoimmunity is the leading symptom in a variety of monogenic disorders that affect T cell development, tolerance, and interferon signaling, complement pathways as well as the resolution of inflammation, this view is changing nowadays (157). The two prototypic examples to illustrate their close interrelationship are the autoimmune lymphoproliferative syndrome (ALPS) and the IPEX syndrome (immune dysregulation, polyendocrinopathy, enteropathy, X-linked). In case of ALPS, the respective lymphoproliferation and propensity to develop lymphomas results from apoptosis-impairing germline as well as somatic mutations in the *FAS, FASL*, and caspase 10 genes (160–162). Still, mutation carriers have only a <60% chance for disease manifestation (161). The IPEX syndrome, on the other hand, is caused by activating mutations in the *FOXP3* gene (163–165). The encoded transcription factor controls the function of regulatory T cells that are essential for maintaining self-tolerance and immune homeostasis by suppressing aberrant responses such as autoimmunity and allergy (166). Deficiency of cytotoxic T lymphocyte antigen 4 (CTLA-4), which is a crucial inhibitor of T cell response that is also present on regulatory T cells, can therefore generate autoinflammation and autoimmunity in a corresponding fashion (166–169). Moreover, CTLA-4-deficient individuals are at risk to primarily develop EBV-related malignancies (170). Another recently recognized albeit rare cause of autoimmunity are leukemia-predisposing germline mutations in the *IKZF1* gene, which encodes the hematopoietic transcription factor Ikaros (171–175). Finally, one should not forget that autoimmune diseases are also a common problem in one of the most remarkable forms of leukemia-predisposing immunodeficiencies, the Down syndrome (176–178).

### Hyperactivation

The inability of cytotoxic lymphocytes to fend off and kill virus-infected or transformed cells leads to an often uncontrollable hyperactivation of the immune system in form of hemophagocytic lymphohistiocytosis (HLH) (179–181). This distinctive clinical feature is the common denominator of a related group of perforin-deficient hyperinflammatory disorders, so called “perforinopathies,” that may either be due to rare congenital gene-disrupting mono- or biallelic mutations or, in less severe form, due to functionally impairing hypomorphic alleles (182–186). Familial hemophagocytic lymphohistiocytosis type 2 (FHL2) is caused by biallelic mutations of the perforin gene (*PRF1*) (179). It shares some of its clinical characteristics with those of anaplastic large cell lymphoma (ALCL), which accounts for ~10 to 15% of all pediatric non-Hodgkin lymphomas (187–190). About a quarter of these lymphoma patients carry only monoallelic *PRF1* mutations but, remarkably, virtually none in *SH2D1A* or *UNC13D*, genes that are implicated in two other forms of FHL (181, 187, 191–193). Moreover, an otherwise common activity-diminishing *PRF1* gene variant (SNP A91V; rs35947132), is also postulated to predispose to the nasal form of NK/T cell lymphoma in adults, which is the most frequent EBV-related NK/T cell malignancy (188).

### Microbiome

Human beings are holobiontic meta-organisms (194–197). They are composed of host as well as trillions of viral, fungal, bacterial, and eukaryotic microbes that are collectively referred to as the microbiota or microbiome (194–197). This microbiome is acquired and shaped during the first 2 years of life. It co-evolves with its respective host genome and, under physiological conditions, becomes part of a stable, life-long synergistic homoeostasis (194, 195, 197–200). Because of its tight functional link with and profound effects on the host's immune system in health and disease, the microbiome is therefore already regarded as a complex, polygenic trait (200–202). Environmental, and host-related perturbations of this microbial ecosystem reduce almost invariably its diversity. This is a common finding in many multifactorial inflammatory, autoimmune, metabolic, neoplastic, and neurodegenerative diseases, although it is rarely known whether such a dysbiosis is indeed the cause or the effect of the underlying ailment (197, 203). Nevertheless, the host's immune system is certainly the most important force that shapes the configuration of the normal and dysbiotic microbiome, which, in turn, may of course be a significant cofounding factor in immune-mediated and immune-associated diseases (197). A healthy or dysbiotic microbiota can influence the host innate immune system and it is therefore no surprise that microorganisms are also implicated in the pathogenesis of at least 20% of all human malignancies (204). In a dysbiotic state, alterations in the signature of microbial molecules that are sensed by the host can lead to a different activation state of the immune system. These changes may alter the balance of host cell proliferation and death, guide immune system function and influence metabolism of host-produced factors, ingested food and pharmaceuticals (205). Moreover, they may also drive transformation by affecting genomic stability, resistance to cell death and proliferative signaling (205). Both chronic high-grade as well as lower-grade smoldering inflammatory disorders drive a tumor-permissive milieu, a problem that was extensively studied and confirmed in mice that were deficient in various immunologically relevant genes (199, 205, 206). It is worth noting that such a cancer susceptibility can even be transferred to healthy mice by cohousing, fostering or fecal transplants (195, 199, 207). Since polymorphisms in immunologically relevant genes affect human microbiota composition and cancer predisposition, this observation is therefore also highly relevant for such human diseases (201, 202, 208, 209).

Based on these results, Dzutsev et al. therefore suggested that malignancies can be viewed as systemic diseases that alter the physiological homeostatic interaction of the entire meta-organism (143, 195). Mostly because of its effects on metabolism, cellular proliferation, inflammation, and immunity, the microbiome would interact with their development at virtually every level, including predisposing conditions, initiation, genetic instability, susceptibility to host immune response, progression, comorbidity and, not least, response to therapy (143, 195, 205). In support of this notion, Yamamoto et al reported, for instance, that variation in intestinal microbes between different animal facilities or as a consequence of experimental perturbations profoundly affected the incidence of lymphoma and survival of Atm (ataxia telangiectasia mutated)-deficient mice (210). Another very intriguing and instructive example of how we will one day perhaps be able to exploit particular constituents of the microbiome for medical and therapeutic purposes was recently provided by Bromberg et al. (211). They showed that with the delivery of stool samples from pregnant mice or gavage with isolated B. pseudolongum species to those with cardiac allografts they were able to improve their long-term survival as well as to prevent inflammation and fibrosis in this respective organ (211).

### *Bona Fide* Infections

The IARC classifies 10 microbial agents (7 viruses, 2 parasites, and 1 bacterium) as group 1 human carcinogens (195, 204). Over 90% of all infection-attributed cancers are attributed to *Helicobacter pylori*, HBV and HCV, and human papillomaviruses (HPV) (204). Except for HCV, all human oncogenic viruses encode at least one oncogene and may therefore directly induce neoplastic transformation, although, as alluded to above, infection-associated inflammation and dysbiosis most likely play a likewise significant role in this context. Thus, Plottel et al. distinguish three classes of microbe-induced human malignancies, the first is defined as involving immunologic tissues, the second requires direct microbial interactions with parenchymal cells and the third involves distant effects from local interactions (212).

The likelihood to be either protected or to become infected as well as the infection outcome depends primarily on the diverse conditions that guide the interactions between the respective pathogen and its potential host. These include their specific genetic set-up and functional fitness to invade or defend themselves, as well as on a variety of other factors, such as concomitant infections as well as the age and microbial constitution of the respective host (213–220). To invade host cells, pathogens exploit and hijack particular cell surface proteins. Amongst the docking receptors that were identified so far in case of the Malaria parasite plasmodium falciparum, for instance, are CD55 and structural variants of the GYPA and GYPB genes (220–224). CD55-null erythrocytes, in particular, are refractory to invasion by all isolates of plasmodium falciparum (222). The second example is *CCR5*, which encodes a coreceptor for HIV entry. Consequently, carriers of an otherwise phenotypically and functionally completely inconsequential homozygous 32-bp deletion are resistant to HIV infection (225). Although HIV infections are indisputably the cause of the “acquired” immune deficiency, one can still argue that all these infection-related problems are nevertheless due to a genetically primed primary albeit clinically inapparent susceptibility. The point I want to stress here is that what one currently perceives as a “normal” wild-type or a “defective” susceptible gene variant is merely a matter of choice, frequency, common habit, and/or subjective interpretation. Since the functional consequences of any such variant will always remain context-dependent, one therefore needs to keep in mind that the distinction between “protective” and “defective” can never be an absolute dogma, but always lies in the eyes of the beholder.

Epstein-Barr virus (EBV) is a ubiquitous virus that infects virtually all humans and obligatory leads to a usually asymptomatic symbiotic lifelong latent persistence (214, 226). Although this EBV latency may provide an evolutionary mutualistic benefit to its hosts as an immune adjuvant that apparently protects against lethal Listeria monocytogenes and *Yersinia pestis* infections (227, 228), the many EBV-associated problems have nowadays become clinically far more relevant and interesting. At present, the literature distinguishes more than 25 EBV-related disease entities, including those that are associated with various forms of immunodeficiencies and those that concur with a high propensity to develop diverse hematopoietic, epithelial, and mesenchymal malignancies (66, 226, 229–234). These disease forms can be roughly divided into reactive EBV-associated lymphoid and histiocytic/dendritic proliferations (including reactive lesions with or without diverse malignant potential), B cell proliferations (including Hodgkin lymphoma and plasma cell neoplasms), T/NK cell proliferations, immunodeficiency-related lymphoid proliferations and histiocytic/dendritic proliferations (66, 226, 229, 231–236). Taken together, EBV contributes to about 1.5% of all cases of human cancer worldwide (229, 237).

The type and incidence of EBV associated diseases varies significantly in different parts of the world, an observation that can be attributed to the different distribution of genetic susceptibility factors, including individual-, HLA-, and ethnicity-specific ones, to environmental- and geographic-specific co-founding influences but also to the existence of particular EBV strains that may produce different disease patterns (27, 66, 153, 213, 214, 217, 218, 229, 230, 232–234, 236, 238–240).

In contrast to the above-mentioned HIV infection, which significantly increases the risk for malignant diseases and especially lymphomas, such a risk is, if at all, by far not as pronounced in case of Malaria (241–243). The only notable exception concerns the concomitant early and sustained infection of Plasmodium falciparum and EBV, which together are the essential pathogenetic ingredients in the endemic form of Burkitt lymphoma in Africa (217–219, 236, 238, 244–246). In this particular combination, the Plasmodium infection destabilizes the genome of rapidly dividing EBV-infested germinal center B cells by eliciting the protracted expression of the activation-induced cytidine deaminase, a mutation-aggravating enzyme (238, 245).

At one point in their life, virtually all humans become infected with EBV, most of them without any acute, severe or lasting health problems. However, in those who do, one can often identify an underlying disease-associated, more or less pronounced genetic susceptibility, which begs for the question whether EBV-associated disease processes indeed afflict also “immunocompetent” individuals, or put the other way around, what in the end will define such an “immune (in)competence” (235).

## Predisposition to Hematologic Neoplasms in Children

The role of predisposing germline mutations and sequence variants in children and adults with various types of hematologic malignancies was hitherto largely underappreciated, because not all of them concur with nor create any easily recognizable clinical stigmata or suspicious family history. Especially if one screens neoplastic tissues for disease- and/or therapy relevant somatic mutations, it becomes of critical importance to distinguish those from germline ones, because the latter may also have a profound clinical impact as regards choice of therapy, donor selection in case of transplantations, evaluation of comorbidities as well as surveillance strategies (8, 173).

A recent paper by Duan et al. provides an excellent and very comprehensive overview about all the primary immunodeficiencies that in particular predispose to the development of various types of lymphomas and hematologic malignancies (3). The authors compiled more than 60 conditions, which comprised all subgroups of syndromic and non-syndromic cellular and humoral PID as well as defects in phagocytes and innate immunity.

To pack some of the underlying principles and problems into a practical and newsworthy perspective, I will now briefly turn to recent findings in three categories of childhood leukemia.

### Constitutional Trisomy 21

Trisomy 21 is not only the most common chromosome abnormality in liveborns but in many aspects also one of the most outstanding and fascinating examples of an immune system disorder, although for a long time the precise nature of the respective immunological derangements remained elusive (178). Since it is not a monogenic ailment, it is also hardly ever viewed as a primary immunodeficiency, although it clearly concurs with multiple distinct immunological and developmental defects that affect the myeloid but also the early and committed B-lymphoid progenitor compartments in second trimester fetal liver (247). I chose this most intriguing and highly instructive example to explain in which way the predisposing risk score of a kind of “polygenic” disposition can easily reach at least the same magnitude as the one that is otherwise only achievable in a monogenic setting (55).The ensuing variable and clinically often inapparent immunological alterations in Down individuals comprise a mild to moderate decrease in T and B cells, impaired mitogen-induced T cell proliferation, reduced specific antibody responses to immunizations as well as defects of neutrophil chemotaxis (248). Affected individuals suffer from various types of autoimmune and autoinflammation diseases, whereas it is still a matter of debate whether they are also more prone to experience more or severer infections than non-Down individuals (176, 178, 248, 249). The first clues that helped to resolve the functional consequences of this immunological conundrum derived from recent transcriptome and proteome analyses. They revealed that the presence of an extra chromosome 21 leads, amongst others, to an overexpression of the four chromosome 21-encoded interferon receptors and, therefore, place this syndrome into the class of interferonopathies. Interferons are normally produced by cells in response to viral or bacterial infections, regulate genes in neighboring cells and shut down the production of proteins, which activate the immune system and thereby prevent the spread of the infection (250, 251). In line with interferonopathies and autoinflammatory conditions, individuals with Down syndrome display higher levels of many pro-inflammatory cytokines (including IL-6, IL-22, TNF-α, and MCP-1) as well as complement consumption, a state that indicates that the immune system is constantly fighting viral infections that are in fact not there (250). Whether and to which extent such a faulty overreaction may also participate in promoting the development of hematologic neoplasms perhaps in a similar fashion as in genuine virus infection-triggered malignancies, remains to be elucidated.

Individuals with an inborn trisomy 21 have also an extraordinary risk to acquire special forms of hematologic neoplasms in early life, whereas they are otherwise exquisitely protected against the development of any other malignancies (252, 253). Compared to normal age-matched children, the self-limiting transient myeloproliferative disorder (TMD) together with the acute megakaryoblastic leukemia (AML-M7), is ~150 times and the B cell precursor ALL ~33 times more common (99, 101, 102, 253, 254). Of particular note are also the absence of infant ALL, the rarity of T-ALL and, compared to normal children, the different distribution pattern of genetic B-ALL subtypes (255).

The occurrence of specific mutations in the receptive precursor cells determine which form of leukemia will eventually develop. In case of TMD, the perturbation of megakaryocyte-erythroid precursor cell differentiation fosters the appearance of a highly specific truncating mutation in exon 2 of the hematopoietic transcription factor GATA1. This in turn provides the receptive cellular and molecular environment for the occurrence of further mutations, primarily in the JAK and RAS signaling pathways as well as in epigenetic regulators and multiple cohesion components, which then facilitate the further progression into AML (99, 101, 104, 254). A reduced lymphoid gene expression in fetal liver hematopoietic precursor cells impairs B-lymphoid development in a similar fashion. The ensuing maturation arrest leads to an ~10-fold reduction in B cells. The concomitant accumulation of pro-B progenitors (247), on the other hand, increases the likelihood for illegitimate V(D)J recombination-mediated chromosomal rearrangements, in particular *CRLF2* gene fusions, that can be found in approximately half of all Down syndrome BCP ALL cases (99, 104). To explore the potential contribution of chromosome 21-encoded and overexpressed genes, a set of 31 triplicated orthologous human genes were tested in germline mouse models (256). Their presence induced progenitor B cell self-renewal *in vitro*, maturation defects *in vivo* and the development of especially *CRLF2*-rearranged and *JAK2* pathway-activated BCP ALL. Out of these 31 genes, the nucleosome-remodeling protein high mobility group nucleosome-binding domain- containing protein 1 (*HMGN1*), whose protein product suppresses H3K27me3, turned out to be the most relevant candidate. Together with secondary alterations in the *CRLF2, JAK2, NRAS*, or *KRAS* genes, it promotes both B cell proliferation *in vitro* and the development of B ALL in mice *in vivo* (256).

Given the extraordinary susceptibility and the high incidence of leukemias, one would intuitively expect that both myeloid and lymphoid forms should occasionally occur together by pure chance alone. However, such a coincidence has so far never been reported. On the one hand, this lack of co-occurrence might indicate that the development of a specific type of leukemia requires and is subject to very individual-specific predisposing conditions. On the other hand, it is also in keeping with the fact that these patients virtually never suffer from secondary neoplasms and that they are in a unique and matchless way also protected against the development of any other types of neoplasms (252, 253).

This protective effect also has been put down to a copy number-dependent gene dosage but also context-dependent effect of specific chromosome 21-encoded genes. The presence of three *ETS2* copies, for instance, act as tumor repressor in the ApcMin intestinal cancer mouse model, whereas in the PyMT breast cancer mouse model it functions as tumor promoter, albeit within the non-cancerous stromal cells, where it regulates the expression of genes that produce the extracellular matrix, an essential component for tumor growth and metastasis (257–260). Two other relevant genes are the Down's syndrome candidate region-1 (*DSCR1*), which encodes a suppressor of the vascular endothelial growth factor (VEGF)-mediated angiogenic signaling by the calcineurin pathway, and *DYRK1A*, which encodes a regulator of cell proliferation (261). In mice, the presence of a single extra copy of Dscr1 is sufficient to diminish tumor growth by suppressing the calcineurin pathway and therefore also angiogenesis, an effect that is significantly enhanced by an extra copy of Dyrk1a. For the sake of completeness, one needs to take at least also note of several other trisomic chromosome21-encoded genes whose presence in the stromal compartment helps to reduce tumor angiogenesis, namely the angiogenic inhibitor *ADAMTS1*, the transcription regulator *ERG* and, finally, the endothelial cell-specific genes *JAMB* and *PTTG1IP* (257).

### Bone Marrow Failure (BMF), Myelodysplastic Syndromes (MDS), and Myeloid Leukemias

Table 1 provides a comprehensive summary of the various disease entities together with their causative genetic background. For a more in-depth overview I refer the interested reader to recent publications that deal with these individual subjects in detail (8, 60, 158, 264, 265, 268, 269, 272, 275, 283, 299, 300). Herein, I merely select a few instructive examples to highlight some of the intriguing phenomena that are particularly pertinent for the topic discussed herein.

**Table 1:** Immunodeficiency syndromes that predispose to the development of bone marrow failure, myelodysplasia and myeloid leukemias.

**Disease/Syndrome**	**Defective genes**	**Malignancy risk**	**Remarks**	**References**
Fanconi Anemia (FA)	*FANCA, FANCB, FANCC, FANCD1/BRCA2, FAND2, FANCE, FANCF, FANCG/XRCC9, FANCI/KIAA1794, FANCJ/BRIP1/BACH1, FANCL, FANCM, FANCN/PALB2, FANCO/RAD51C, FANCP/SLX4, FANCQ/ERCC4, FANCR/RAD51, FANCS/BRCA1, FANCT/UBE2T, FANCU/XRCC2, FANCV/REV7/MAD2L2*	MDS, AML, T-ALL, squamous cell carcinomas (head & neck, genitourinary tract), breast cancer	Currently 21 known genes that encode members of the FA/BRCA repair complex	(8, 262–265)
**RIBOSOMOPATHIES**				
Diamond_Blackfan anemia (DBA)	*RPL3, RPL5, RPL10, RPL10A, RPL11, RPL15, RPL18, RPL19, RPL26, RPL27, RPL31, RPL34, RPL35, RPL35A, RPS7, RPS10, RPS11, RPS15A RPS17, RPS19, RPS24, RPS26, RPS27, RPS28, RPS29, RPLP0, TSR2, GATA1, CECR1*	25% life-long overall risk of 5.4 odds ratio MDS, AML, colon cancer, osteogenic sarcoma, and genital cancer	26 ribosomal genes, 6% phenocopies in non-ribosomal genes, 22% unidentified	(265–269)
Dyskeratosis Congenita (DC)	*ACD, CTC1, DKC1, NAF1, NHP2, NOP10, PARN, POT1, RTEL1, TERC, TERT, TINF2, WRAP53, STN1/OBFC1*	MDS, AML, squamous cell cancers of the head, neck & anogenital region	Telomere-associated ribonucleoprotein (RNP) and shelterin complexes	(265, 269–272)
Shwachman-Diamond-Bodian Syndrome (SDBS),	*SBDS, DNAJC21/HSP40, EFL1*	5% leukemia risk (AML, CML, ALL)	Defective processing of rRNA into ribosome assembly, majority unidentified	(265, 269, 273)
Cartilage hair hypoplasia (CHH)	R*MPR*	Non-Hodgkin lymphoma, basal cell carcinoma	RNA component of RNAse MPR, one single Finnish founder mutation	(265, 269, 274)
Aplastic anemia/pancytopenia	*MECOM, ERCC6L2*	MDS		(275)
**PLATELET DISORDERS**				
Amegakaryocytic thrombocytopenia	Mostly *MPL* (thrombopoietin receptor), *RUNX1, ANKRD26, MYH9, PTPN1*	Pancytopenia, leukemia		(265, 276)
Thrombocytopenia absent radius (TAR) syndrome	del(1q21.1) & *RBM8A* SNP	Leukemia (rare)		(265, 277)
Familial thrombocytopenia	*ETV6, RUNX1, DDX41, ANKRD26*	MDS, leukemias		(8)
Congenital neutropenia	*CSF3R, ELANE, G6PC3, GFI1, HAX1, JAGN1, VPS45, WAS*	G-CSF treatment, dose-dependent MDS/AML risk		(264, 278, 279)
*GATA2* deficiency (Emberger & Monomac syndrome)	*GATA2*	MDS, AML (monosomy 7)		(275, 280–286)
Mirage & ataxia-pancytopenia syndrome	*SAMD9, SAMD9L*	MDS, AML (monosomy 7)		(273, 287–294)
Rasopathies	*NF1, PTPN11, CBL, NRAS, KRAS*	JMML, ALL		(295–298)

*Apart of being present in the germ line, somatic mutations of most of these genes can be commonly encountered in sporadic forms of analogous malignancies*.

Fanconi Anemia (FA) is not only the most common inherited BMF disorder but, with a 500- to 700-fold higher incidence of head and neck squamous cell carcinomas in older patients, also a highly penetrant cancer susceptibility syndrome (301). Except for the X-linked *FANCB* gene, it is due to bi-allelic mutations that can affect one of 21 genes, which encode various components of the evolutionarily conserved FA/BRCA repair complex. Five of these (*BRCA2, PALB2, RAD51C, SLX4*, and *BACH1*) specifically predispose also to breast cancer. The encoded proteins participate in biochemical pathways that safeguard not only against the effects of alkylating agents and radiation but, probably even more relevant, also against those of endogenous aldehydes, oxidative stress, inflammation, mitophagy, and virophagy (263, 265, 302, 303). All these factors damage DNA in form of distinct inter-strand DNA crosslinks. The inability to repair these damages is the primary driver of the various biological and clinical problems that define this disease category.

Small aldehydes, such as acetaldehyde and formaldehyde, are not only ubiquitously present in the environment, but also potentially toxic byproducts of the normal cellular metabolism and especially de-methylation reactions (304–306). Given that they provide already a rich source for endogenous inter-strand DNA and protein cross links, one can envisage that the pathogenic manifestations and consequences of FA mutations may also be strongly influenced and modified by the functional capability of aldehyde detoxifying enzymes, such as aldehyde dehydrogenase 2 (ALDH2) and alcohol dehydrogenase 5 (ADH5) (306, 307). In line with this notion, Japanese FA children that carried a functionally deficient ALDH2E504K allele were shown to progress more rapidly to aplastic anemia but not to MDS or AML (307–309). Moreover, malformations were only more severe in two of three homozygous carriers, which indicates that a deficient maternal genetic background might contribute to this outcome (310). Maternal-produced aldehydes diffuse indeed across the placenta and can thus damage the developing embryo's DNA, whereas embryo-derived ones can in turn be detoxified by the maternal organism. An inappropriate *in utero* exposure, such as an excessive maternal ethanol consumption during gestation, would therefore aggravate not only the formation of congenital abnormalities in FA but it provides also an intriguing etiological link to analogous phenotypic changes that define the alcohol embryopathy (308). Whether a disturbed aldehyde detoxifying system might also be causatively involved in the *in utero* initiation of childhood leukemias remains currently a matter of speculation (307).

In addition to stalling and destabilizing DNA replication forks directly, formaldehyde also selectively depletes BRCA2 via proteasomal degradation, a circumstance that poses a special risk for heterozygous *BRCA2* mutation carriers. In these, formaldehyde-induced degradation can decrease the respective protein levels below the otherwise protective one of normal wild-type individuals and thereby potentiate their mutagenic vulnerability (311).

Taken together, these observations have significant implications for risk awareness and avoidance as well as the clinical management of FA patients. On the positive side, they offer new treatment opportunities, for instance in form of ALDH2 agonists and the widely used diabetes drug metformin, which acts as aldehyde scavenger. At least in mouse models, both of them are able to delay the onset of BMF and malignancies as well as improve hematopoiesis (263, 312, 313).

One remarkable feature of many heritable diseases is that somatic mutations can occasionally autocorrect the particular inherited gene defect in the respective cells (314). Such reverting mutations transform a homozygous or combined heterozygous state again back into a heterozygous functionally compensated state, either through somatic recombination, gene conversion or a compensatory mutation (314, 315). Although this phenomenon is well-known in immunodeficiencies, its effects are most probably still underappreciated. In BMF syndromes such an autocorrection can spontaneously improve or even resolve the specific underlying hematopoietic problem. Amongst others, such spontaneous remissions have repeatedly been documented in FA, dyskeratosis congenita, Diamond-Blackfan anemia, Shwachman-Diamond syndrome and, more recently, in the *SAMD9*- and *SAMD9L*-associated Mirage and ataxia-pancytopenia syndromes, respectively (273, 287–293, 301, 316). Heterozygous *SAMD9L* gain-of-function mutations decrease cell proliferation. The loss of the mutation-carrying chromosome 7 in bone marrow cells therefore leads to the development of MDS and acute myeloid leukemias, whereas a compensating duplication of the normal allele in form of an uniparental disomy (UPD) 7 or 7q is able to resolve the cytopenias (287–293).

The somatic appearance of a complete or partial UPD always draws attention to regions that are usually highly relevant for specific disease processes (317, 318). Such UPDs may either contain duplicated gain of function mutations or, as alluded to in the example above, eliminate them (317, 318). A similar important consequence is the mere transformation of a heterozygous to a homozygous state. In case of the HLA-containing region on the short arm of chromosome 6, for instance, it is clearly exerted through a selective pressure, since the loss of one HLA haplotype is an important immune-escape mechanism. It protects neoplastic cells from the immune surveillance machinery and therefore also plays a crucial role for disease recurrence after haploidentical stem cell transplantations (319, 320). Contrariwise, such a haplotype loss is able to shield hematopoietic cells from the destructive effects of autoimmunity, as has been demonstrated in case of aplastic anemia (321). The practical problems that arise from such a hematopoietic revertant mosaicism is that it may lead to an ascertainment bias and cause difficulties in identifying underlying disease-relevant mutations (322).

The formation of such well-adapted clones might suggest that such compensatory mechanisms are rare events. However, there is ample evidence that this is definitely not the case. Rather than being a life-long stable system the genome is a highly dynamic one that is continuously modified and shaped by ongoing mutational processes, which eventually promote the appearance of cell clones and populations with an increased survival fitness. The formation of somatic mosaicism is therefore the rule rather than the exception, as exemplified by Davis et al., who observed a remarkable clonal heterogeneity and diversity of lymphocytes in a patient with a Wiskott-Aldrich syndrome (323). The continuous generation of such cellular variants and the selective pressures they are exposed to is thus not only a characteristic of the exceptional dynamics of neoplastic but also of normal cells populations (324).

### B-Cell Precursor Acute Lymphoblastic Leukemia (BCP ALL)

Germline lesions that predispose to BCP ALL in children comprise not only those which cause various cancer prone and chromosomal syndromes but also other genuine gene disrupting defects as well as high and low risk variants. The majority of these affect genes that encode B-cell development and transcription factors as well as components of various signal transduction pathways (Table 2) (42, 297, 342).

**Table 2:** Chromosomal locations of GWAS-verified SNPs or genuine germline gene defects that predispose to the development of particular types of childhood ALL.

**Chromosome region**	**Candidate genes**	**Type**	**Function**	**All subset**	**References**
2(q22.3)	Not specified	SNP	–	*ETV6–RUNX1*	(24)
3(q28)	*TP63*	SNP	P53 family of transcription factors	*ETV6-RUNX1*	(325)
7(p12.2)	*IKZF1*	SNP, gene defects	Ikaros family of Zinc finger transcription factors	Not specified	(32, 172, 326, 327)
8(q24.1)	MYC?	SNP	Proto-oncogene, BHLH transcription factor	Not specified	(24, 25)
9(p12)	*PAX5*	Gene defects	Paired box transcription factor	Not specified	(48, 328)
9(p21.3)	*CDKN2A & CDKN2B*	SNP	Cyclin-dependent kinase Inhibitors	Not specified	(329–331)
9(p24.1)	*JAK2*	SNP	Tyrosine kinase	*BCR-ABL1*-like	(49)
10(p12.2)	*PIP4K2A*	SNP	Kinase	Not specified	(329, 332)
10(p14)	*GATA3*	SNP	GATA family of transcription factors	*BCR-ABL1*-like	(332, 333)
10(q21.2)	*ARID5B*	SNP	Transcription coactivator	Hyperdiploid	(26, 32, 326, 327)
10(q26.13)	*LHPP*	SNP	Phosphatase	Not specified	(28)
11(p11.2)	*PTPRJ*	SNP	Family of protein tyrosine phosphatases	*ETV6-RUNX1*	(325)
12(p13.2)	*ETV6*	Gene defects	Proto-oncogene, ETS domain family of transcription factor	Hyperdiploid	(334–338)
12(q23.1)	*ELK3*	SNP	ETS domain family of transcription factor	Not specified	(28)
12(q24.1)	*PTPN11*	Gene defects^*^	Family of protein tyrosine phosphatases	Hyperdiploid	(297)
14(q11.2)	*CEBPE*	SNP	bZIP transcription factor	Hyperdiploid	(23, 25, 32, 327)
16(p13.3)	*CREBBP*	Gene defects^**^	Histone acetyltransferase	Hyperdiploid	Haas, unpublished observation
17(p13.1)	*TP53*	Gene defects^***^	Tumor-suppressor, transcription factor	Hypodiploid	(339–341)
17(q12)	*IKZF3*	SNP	Ikaros family of Zinc finger transcription factors	Not specified	(25)
17(q21.2)	*STAT3*	SNP	Signal transducer and transcription activator	*BCR-ABL1*-like	(49)

For a more general overview about ALL predisposition syndromes and ALL predisposing RASopathies see Kratz et al. and Cave et al., respectively (62, 243).

* *Noonan syndromes, Rasopathy*,

** *Rubinstein-Taybi syndrome*,

*** *Li-Fraumeni syndrome*.

The development of B lymphocytes, in particular, is coordinated by specific regulatory transcription networks that activate the respective B-lymphoid program and at the same time oppress alternate cell fates (343). Somatic mutations in several of these key regulators, such as *IKZF1, TCF3, EBF1*, and *PAX5*, induce leukemic transformation by blocking normal B cell differentiation, which then leads to the accumulation of leukemic B-cell precursors. Although their role in leukemogenesis has been known and explored already for quite some time, the awareness that otherwise apparently inconsequential germline variants may also exert a predisposing effect is quite new. The biological relevance and functional consequences of some of these variants has been confirmed in the meantime with appropriate *in vitro* and *in vivo* experiments and test systems (23–26, 28, 332, 333).

Take for instance *PAX5*, a member of the “paired box” family of transcription factors, which encodes the B cell lineage specific activator protein that is expressed at early but not late stages of B-cell differentiation (195). Out of the three up to now reported families with highly penetrant germline variants, 13 carriers developed ALL (48, 328). In the two families, in which the respective information was provided, all unaffected carriers as well as those with ALL had normal immunoglobulin levels and no evidence of an impaired B cell function at diagnosis (328). Moreover, in line with Greaves two step model, leukemia developed in mice only after exposure to common pathogens and the acquisition of second hits in the IL7R/JAK3/STAT5 signaling axis (344).

Another revealing example is *IKZF1*, which encodes IKAROS, a member of a hematopoietic zinc-finger transcription factor family (345–347). The mutational spectrum of human IKZF1-associated diseases ranges from somatic to germline and from haploinsufficient to dominant negative forms (171). Somatic mutations occur in overall 15% of BCP-ALL and especially in prognostic adverse genetic subtypes (348, 349). Heterozygous germline mutations cause two different forms of immunodeficiency. The haploinsufficient, autosomal dominant late onset form of common variable immunodeficiency (CVID) has an incomplete penetrance, is clinically mild and concurs with a marked decrease in B-cell numbers and immunoglobulin levels as well as autoimmunity (175, 350, 351). The early-onset dominant negative CVID, on the other hand, is characterized by innate and adaptive immune defects of the B, T and myeloid cell lineage (171). Notably, 2/29 of patients with the late onset form developed B-ALL and 1/7 patients as well as another independently reported one with the early onset form developed T-ALL (171, 346, 351). Based on these observations, Churchman and colleagues screened remission samples from 4,963 childhood ALL cases, identified a total of 28 unique *IKZF1* variants in 43 and succeeded to prove a functional consequence in 22 of them (172, 173). Evans et al. even attempted to elucidate the influence of a parental environmental exposure on such leukemia-predisposing risk alleles (352). They found some preliminary albeit hitherto unexplainable evidence that the *IKZF1* risk genotype might have a stronger effect if the mother took folic acid or if the father did not smoke prior to pregnancy (352).

Finally, Auer et al reported a first intriguing example of a “double-hit one-pathway” scenario, in which the biparental inherited combination of two rare germline variants, JAK2 (G571S) and STAT3 (K370R), whose products synergistically interact in the same disease-relevant JAK/STAT signal transduction pathway, is obviously sufficient to induce a Ph-like BCP-ALL (49).

## Concluding Remarks

During their entire development and ongoing existence, both the immune system as well as malignant diseases need to adapt themselves to highly variable and continuously fluctuating environmental conditions, which requires a high flexibility that is largely driven by a combination of interacting antagonistic as well as synergistic deterministic events and regulatory probabilities. “Immunodeficiency” and “tumor susceptibility” are thus two closely intertwined concepts, whose original understanding was based on easily explicable clinical symptoms as well as certain genetic norms. As long as these were based on such more or less simple phenotypic and genotypic features, one did not require any further explanatory definitions. However, the switch from phenotypic to genetic ascertainment programs include now much less obvious disease categories, healthy carriers as well as only vaguely defined potentially predisposed individuals. Although this approach enables of course unprecedented insights into the fine-scale structural and regulatory organization of biological system, the boundaries of classification standards get increasingly blurred, which goes hand in hand with the awareness that it becomes increasingly difficult and virtually impossible to define either of these terms in an unambiguous manner anymore. The more closely one looks, the harder it gets to find genes that are not in one or the other way part of this game.

In his highly recommendable and readable book “Tending Adam's garden,” Irun Cohen portrays the immune system as a cognitive system (353). Like its prototypic equivalent, the brain, it learns through individual experience and thereby forms a functionally highly efficient, flexible, and to some extent also redundant interactive structure. As Cohen pointed out, a particular gene may only become essential once the system has organized itself around it so that thereafter the system becomes dependent on it. In case this particular gene is already defective at the beginning, the system often can compensate for this loss and organize itself around an alternative gene, which then becomes the essential one.

Thus, organizational entities depend not only on distinct features of particular sub-units but even more so on their functional interactions. In case of the immune system, such multi-component, self-emergent networks comprise a variety of distinct cellular as well as humoral host constituents, but also a manifold of environmental factors, such as the maternal immune system, the microbiome, infectious agents, as well as physical and chemical agents, that co-govern and modulate, but often also interfere and disrupt its normal development during different stages.

Thus, organizational entities depend perhaps less on the appropriate function of particular genetic sub-units alone but much more on the successful interaction of their cellular and humoral products, an observation that is readily evident in case of genetically determined developmental disorders that can cause both immune system deficiencies as well as malignant diseases.

The essential implication of Cohens' model is that we only may become more successful in curing such disease when we begin to understand the decision-making processes of the immune system rather than that of the effects of individual components alone.

## Author Contributions

The author confirms being the sole contributor of this work and has approved it for publication.

### Conflict of Interest Statement

The author declares that the research was conducted in the absence of any commercial or financial relationships that could be construed as a potential conflict of interest.
